# Evaluation of Rhenium and Technetium-99m Complexes Bearing Quinazoline Derivatives as Potential EGFR Agents

**DOI:** 10.3390/molecules28041786

**Published:** 2023-02-14

**Authors:** Konstantina Makrypidi, Christos Kiritsis, Ioanna Roupa, Sotiria Triantopoulou, Antonio Shegani, Maria Paravatou-Petsotas, Aristeidis Chiotellis, Maria Pelecanou, Minas Papadopoulos, Ioannis Pirmettis

**Affiliations:** 1 Institute of Nuclear and Radiological Sciences and Technology, Energy & Safety, NCSR “Demokritos”, 15310 Athens, Greece; 2 Institute of Biosciences & Applications, NCSR “Demokritos”, 15310 Athens, Greece

**Keywords:** EGFR, TKI, PAMA, cysteine, rhenium, technetium-99m, tricarbonyl

## Abstract

Τhe Epidermal Growth Factor Receptor tyrosine kinase inhibitor (EGFR-TKI) 6-amino-4-[(3-bromophenyl) amino]quinazoline was derivatized with 6-bromohexanoyl-chloride and coupled with the tridentate chelating agents N-(2-pyridylmethyl) aminoethyl acetic acid (PAMA) and *L*(+)-cysteine bearing the donor atom set NNO and SNO, respectively. The rhenium precursors ReBr(CO)_5_ and *fac*-[NEt_4_]_2_[ReBr_3_(CO)_3_] were used for the preparation of the Re complexes *fac*-[Re(NNO)(CO)_3_] (**5a**) and *fac*-[Re(SNO)(CO)_3_] (**7a**) which were characterized by NMR and IR spectroscopies. Subsequently, the new potential EGFR inhibitors were labeled with the *fac*-[^99m^Tc(CO)_3_]^+^ core in high yield and radiochemical purity (>90%) by ligand exchange reaction using the *fac*-[^99m^Tc][Tc(OH_2_)_3_(CO)_3_]^+^ precursor. The radiolabeled complexes were characterized by comparative HPLC analysis with the analogous rhenium (Re) complexes as references. In vitro studies in the A431 cell lines showed that both ligands and Re complexes inhibit A431 cell growth. Complex **5a** demonstrated the highest potency (IC_50_ = 8.85 ± 2.62 μM) and was further assessed for its capacity to inhibit EGFR autophosphorylation, presenting an IC_50_ value of 26.11 nM. Biodistribution studies of the ^99m^Tc complexes in healthy mice showed high in vivo stability for both complexes and fast blood and soft tissue clearance with excretion occurring via the hepatobiliary system.

## 1. Introduction

The epidermal growth factor receptor (EGFR) family (Erb B) consists of four members known as EGFR (ErbB-1 or HER-1), ErbB-2 (or HER-2), ErbB-3 (or HER-3), and ErbB-4 (or HER-4). EGFR is found to be overexpressed and dysregulated in numerous solid tumors. EGFR possesses an extracellular ligand binding domain, a hydrophobic transmembrane region, and an intracellular cytoplasmatic domain with tyrosine kinase (TK) activity. Successful interaction between the extracellular domain and the appropriate natural ligand (e.g., EGF) initiates structural alterations and dimerization of the receptor to homodimers or heterodimers, followed by adenosine triphosphate (ATP) binding in the intracellular tyrosine kinase domain and finally autophosphorylation of EGFR. After the receptor’s autophosphorylation, several downstream signal transduction pathways are triggered and involved in cell proliferation, differentiation, migration, adhesion, and apoptosis. Over-expression of EGFR and its enhanced signaling are associated with uncontrollable proliferation, tumor angiogenesis, cellular invasion and metastasis, and resistance to some therapy methods [1,2,3,4].

The design and development of anticancer agents that can specifically bind to EGFR, inhibiting its TK activity and downstream signaling, has aroused great interest over the years, resulting in the development of several therapeutic approaches targeting EGFR. Monoclonal antibodies compete with the natural EGFR ligands and act in the extracellular domain inhibiting TK activation and autophosphorylation [5]. Many monoclonal antibodies, such as cetuximab, have been used against malignancies (colorectal cancer) and have been tested in clinical trials [6]. Another approach is the development of tyrosine kinase inhibitors (TKIs), small molecules that bind reversibly or irreversibly to the intracellular tyrosine kinase domain. Several types of TKIs have been synthesized and evaluated. Among them, the 4-anilinoquinazoline derivatives are the most potent and selective [7]. Characteristic examples are the quinazoline derivatives gefitinib (Iressa^®^) and erlotinib (Tarceva^®^), which are clinically used against non-small cell lung cancer (NSCLC). Gefitinib and erlotinib bind to the ATP binding site of the enzyme, preventing TK phosphorylation and activating EGFR signal transduction pathways [8].

Imaging EGFR has greatly attracted the attention of the radiopharmaceutical research community as it contributes to the early detection and staging of EGFR-positive tumors and patients’ response to EGFR-targeted therapies. Numerous reversible and irreversible inhibitors, primarily those bearing the 4-anilinoquinazoline analogue, have been labeled with positron emitters, such as ^18^F, ^11^C, ^124^I, and ^68^Ga [9], and their potential as positron emission tomography (PET) imaging agents was evaluated. Nevertheless, technetium-99m (^99m^Tc) remains the most employed radionuclide in nuclear medicine for diagnostic applications with single photon emission tomography (SPECT) due to its near ideal physical properties (t_1/2_ = 6.02 h; E*γ* = 140 keV), low cost and widespread availability, characteristics that still justify the efforts for developing ^99m^Tc-labeled quinazoline derivatives.

In the majority of the reported ^99m^Tc-labeled quinazoline derivatives [10,11,12,13], the 4-anilinoquinazoline pharmacophore was coupled to tailor-made chelators suitable for the stabilization of either the M^III^ (M = Re and ^99m^Tc) core with a ‘‘4+1′’ mixed-ligand system or the tricarbonyl *fac*-[M^I^(CO)_3_]^+^ core. It was shown that the presence of the organometallic core does not deteriorate the pharmacological properties of the 4-anilinoquinazoline pharmacophore. As part of our ongoing efforts to develop EGFR imaging agents, we present herein the synthesis, characterization, and biological evaluation of two novel ^99m^Tc-labeled derivatives that bear the 4-anilinoquinazoline pharmacophore and a suitable tridentate chelating system that stabilizes the *fac-*[M^I^(CO)_3_]^+^ (M = Re/^99m^Tc) moiety. The N-(2-pyridylmethyl) amino acetic acid (PAMA) and *L*-cysteine are used as the tridentate chelating agents as they have the appropriate donor atom system to form neutral, stable hexacoordinated complexes of the type *fac-*[M^I^(NNO)(CO)_3_] and *fac-*[M^I^(SNO)(CO)_3_] (M = Re/^99m^Tc) [14,15,16,17]. As the sixth position of 3-bromo-4-anilinoquinazoline leaves room for structural modifications without abolishing the potency of the pharmacophore scaffold, this work aims to explore the effect on the physicochemical and pharmacological properties of the complexes when the chelator is situated on a remote position compared to the pharmacophore structure. The latter is accomplished by introducing a lengthy alkyl chain between the two moieties.

## 2. Results and Discussion

### 2.1. Chemistry

#### 2.1.1. Synthesis of the Ligands

**Figure 1:**
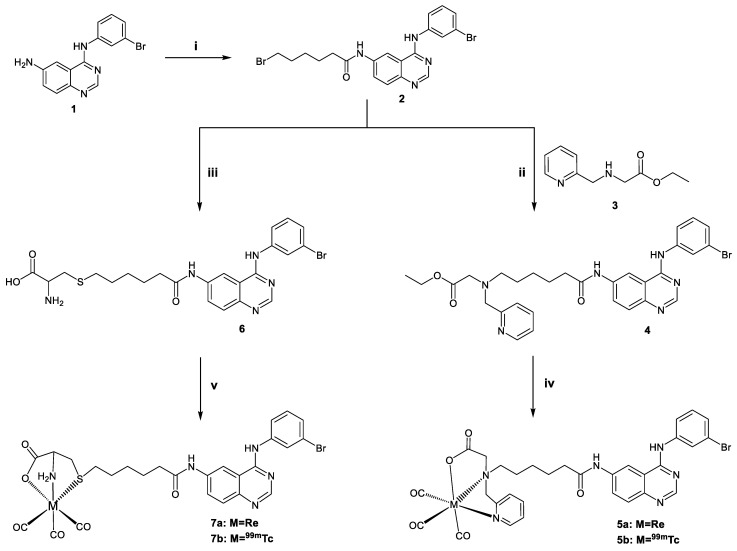
Synthesis of ligands **4**, **6** and complexes **5a**, **5b**, **7a**, **7b**. **i**: 6-bromo hexanoyl chloride, anhydrous THF, 0 °C 30 min, rt for 3 h, **ii**: NaI, DIPEA, THF, CH_3_CN, reflux, 18 h, **iii**: *L*-cysteine, NaOH, EtOH, rt, 18 h, **iv**, and **v**: *fac*-[NEt_4_]_2_[ReBr_3_(CO)_3_] (Method A), [ReBr(CO)_5_] (Method B), *fac*-[^99m^Tc][Tc(OH_2_)_3_(CO)_3_]^+^.

The new tridentate ligands **4** and **6** bear the known tyrosine kinase inhibitor 6-amino-4-[(3-bromophenyl) amino] quinazoline (**1**) and were synthesized via a five-step reaction using the 4-hydroxyquinazoline as starting material. First, **1** was reacted with 6-bromohexanoyl chloride to afford bromide **2** in good yields. Next, the tridentate ligand **4** was prepared by reaction of PAMA (**3**) and **2,** albeit in low yield (20%) (Figure 1). The reaction of **2** with *L*-cysteine under basic conditions afforded tridentate ligand **6** in good yield (70%). The reaction was conducted under a nitrogen atmosphere to avoid cysteine oxidation by forming disulfide bonds.

#### 2.1.2. Synthesis of the Rhenium Complexes

Two new neutral hexacoordinated rhenium tricarbonyl complexes, **5a** and **7a**, of the general form *fac*-[Re(NNO)(CO)_3_] [15,17] and *fac*-[Re(SNO)(CO)_3_] [14,16] with PAMA and cysteine as chelators, respectively, were synthesized. The rhenium complexes were synthesized by reacting equimolar amounts of the corresponding ligands and rhenium precursor. As precursors, either [ReBr(CO)_5_] (Method A) or *fac*-[NEt_4_][ReBr_3_(CO)_3_] (Method B) were used [10]. The synthesis of complex **5a** was performed under basic conditions to enable hydrolysis of the esterified PAMA and generate the active ligand form. Both Methods A and B employed (see Section 3.1 in Material and Methods) demonstrate similar yields (71%, Method A and 78%, Method B) despite the higher reaction time in the first method (18 h, Method A and 3 h, Method B). Complex **7a** was also synthesized by employing the two precursors. The yield in Method A and B was 69 and 52%, respectively.

### 2.2. Spectroscopy Studies

All synthetic compounds were characterized by IR and ^1^H-NMR. In the IR spectrum of derivative (**2**), the presence of new peaks at 2934 and 1675 cm^−1^ compared to starting material **1** indicates the presence of the aliphatic chain and the amide, respectively. In the ^1^H-NMR spectrum, the integration of the peaks and multiplicity of aliphatic and aromatic protons agree with the structure of derivative **2**.

In the IR spectrum of ligand **4**, the characteristic peak at 1732 cm^−1^ corresponds to a v (C=O) vibration, indicating the presence of an ester. The peaks at 2923 and 1669 cm^−1^ correspond to v (C–H) and v (C=O) vibrations of the aliphatic chain and the amide, respectively. In the ^1^H-NMR spectrum, the presence of characteristic peaks of the pyridine protons at 8.45, 7.72, 7.46, and 7.21 ppm denote the presence of PAMA on the structure of **4** as they belong to the pyridine protons.

Similarly, in the IR of ligand **6**, the peak at 3283 cm^−1^ corresponds to the vibration of the primary amine of cysteine, while the peaks at 2940 and 2845 cm^−1^ are attributed to the v (C–H) stretch of the aliphatic chain. In the ^1^H-NMR spectrum, the distinctive peaks of methylene protons cysteine are observed at 3.01 and 2.94 ppm as doublets of doublets.

The characteristic finding in the infrared spectra of complexes **5a** and **7a** is the strong bands in the 2030–1890 cm^−1^ range, attributed to the v (C–O) stretch of the *fac*-[Re(CO)_3_]^+^ unit. In the spectrum of **5a**, the absence of the peak at 1732 cm^−1^, corresponding to the carbonyl ester, indicates the ester hydrolysis. Medium bands at 2933 cm^−1^ (for **5a**) and 2927 cm^−1^ (for **7a**) also indicate the presence of an aliphatic chain which is tethering the chelating agent to the 4-anilinoquinazoline core.

In the ^1^H-NMR spectrum of complex **5a**, the pyridine protons are shifted downfield in agreement with the coordination of the pyridine N to the *fac*-[Re(CO)_3_]^+^ metal core. In addition, the geminal CH_2_ protons of the PAMA ligand—that appear together in the spectrum of the free ligand—are chemically shift differentiated in the spectrum of **5a** and appear as separate doublets, characteristic of complex formation [18,19]. In the ^1^H-NMR spectrum of complex **7a** downfield shift is noted for the α-proton of cysteine compared to free ligand **6**, accompanied by broadening of the β-protons, as typically observed upon SNO coordination of S-substituted cysteine to the *fac*-[Re(CO)_3_]^+^ core [14,16,20]. As expected, the chemical shifts of the quinazoline moiety protons do not show any appreciable changes upon coordination, being far from the metal center.

### 2.3. Radiochemistry

#### Preparation of ^99m^Tc-Complexes

The ^99m^Tc complexes **5b** and **7b** were prepared by ligand exchange reactions using the *fac*-[^99m^Tc][Tc(OH_2_)_3_(CO)_3_]^+^ tricarbonyl complex as the precursor. After the reduction of pertechnetate under a carbon monoxide atmosphere, the tricarbonyl precursor was formed, and the pH of the reaction mixture was adjusted to 5 using HCl 1 N. The reaction was monitored by RP-HPLC. The addition of the *fac*-[^99m^Tc][Tc(OH_2_)_3_(CO)_3_]^+^ into a solution of **4** or **6** in DMSO (concentration in the final labeling solution 10^−4^ M) at 70 °C for 30 min resulted in the formation of the complexes **5b** and **7b**, respectively. HPLC analysis showed the quantitative formation of these two new complexes (90–95%). The structures of the technetium-99m complexes were identified by comparative HPLC applying radiometric and photometric detection simultaneously. Hence, co-injection of complexes **5a** and **5b** or **7a** and **7b** exhibited practically identical retention times (Table 1), indicating that the corresponding technetium-99m and rhenium complexes are of the same chemical structure.

The stability of complexes **5b** and **7b** was studied in their reaction mixture and after isolation by HPLC for up to 24 h. No decomposition was observed either at room temperature or at 37 °C. The complexes **5b** and **7b** also demonstrate high stability (>95%) after 24 h incubation with an excess of histidine and cysteine at a molar ratio of 1/1000, as monitored by HPLC.

The pharmacokinetic profile of the studied compounds could be affected by their lipophilicity, expressed as the logarithm of the distribution coefficient (log D_o/w_). The lipophilicity of complexes **5b** and **7b** was determined in physiological conditions (*n*-octanol/0.1 M phosphate buffer pH 7.4). The results showed that complexes **5b** and **7b** are lipophilic with log D_7.4_ values of 3.27 ± 0.25 and 2.87 ± 0.32, respectively. The presence of the pyridine ring in the chemical structure of complex **5a** increases the log D_7.4_ value, as expected.

### 2.4. Biological Evaluation

Previous studies reported by our team [10,11,13] have demonstrated that the rhenium complexes with 4-anilinoquinazoline derivatives inhibit phosphorylation and demonstrate high cytotoxicity in the human cancer cells (A431 cell line), which overexpress the epidermal growth factor receptor (EGFR). Therefore, the newly synthesized rhenium complexes and ligands bearing the pharmacophore moiety 3-bromo-4-anilinoquinazoline were also evaluated for their cytotoxic properties, while complex **5a**, with the lowest IC_50_ value, was evaluated further for its potency to inhibit EGFR phosphorylation.

#### 2.4.1. Inhibition of Cell Growth (MTT Assay)

**Figure 2:**
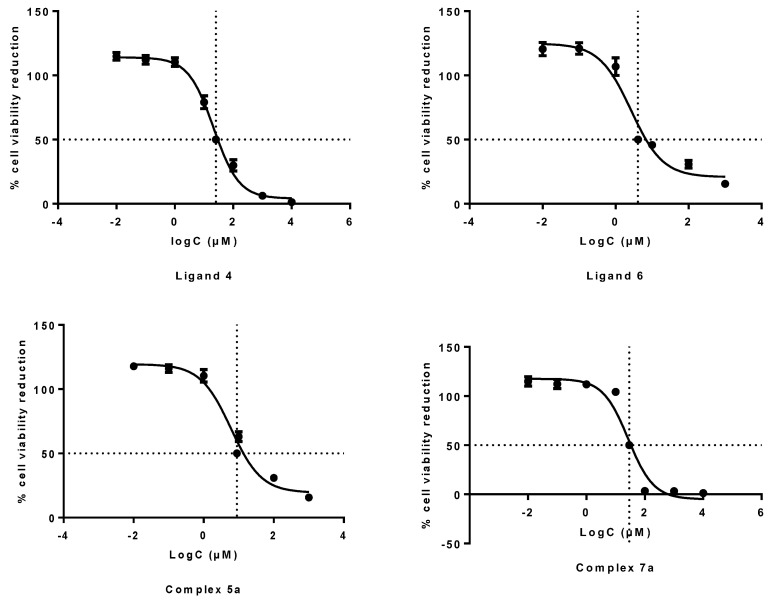
Dose−response curves from MTT assay for the ligands **4**, **6**, and complexes **5a**, **7a** at a concentration range from 1 mM to 10 nΜ.

The cytotoxicity evaluation of compounds **1**, **2**, **4**, **6**, **5a** and **7a** was performed by the MTT assay in A431 cells. This assay measures the amount of MTT reduction by mitochondrial dehydrogenase and assumes that cell viability (corresponding to the reductive activity) is proportional to the production of purple formazan that is measured spectrophotometrically. The obtained results are presented as dose−response curves in Figure 2, while the concentration of each compound that inhibits 50% of cell viability (IC_50_) is presented in Table 2.

Comparing the ligands and their rhenium complexes, ligand **6** exhibited the most significant cytotoxic activity (4.12 ± 0.92 μM), which was decreased after complexation with the tricarbonyl rhenium moiety (**7a**) (29.56 ± 6.56 μM). The complex *fac*-[Re(NNO)(CO)_3_] (**5a**) appeared to be more toxic to the A431 cancer cells as the IC_50_ value of ligand **4** (25.64 ± 6.1 μM) was improved significantly (8.85 ± 2.62 μM) after coordination to the tricarbonyl core. It should be noted that these values are similar to previous MTT experimental results referred to in the literature (Table 3). Amongst the compounds bearing the PAMA chelator, ligand **4** (6-carbon chain) presented a slightly higher IC_50_ value than ligand **8** (23.8 ± 5.1 μM) of Fernandes et al. [10] (2-carbon chain), while in the Re complexes, the reverse effect was observed, and chain expansion from 2 (Fernandes et al.) to 6 carbon atoms (complex **5a**) enhanced the cytotoxicity.

#### 2.4.2. Inhibition of EGFR Phosphorylation

**Figure 3:**
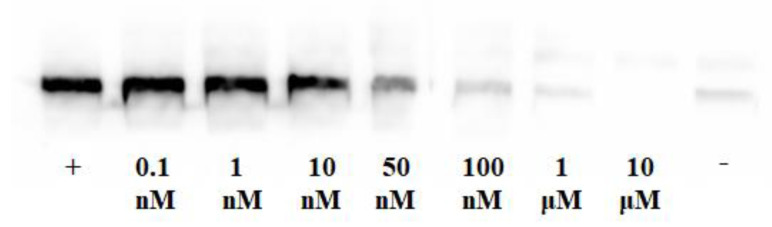
Inhibition of EGFR phosphorylation in A431 cells for complex **5a** at a concentration range from 0.1 nM to 10 μM. Symbols + and- indicate the presence or absence of EGF in the experiment.

**Figure 4:**
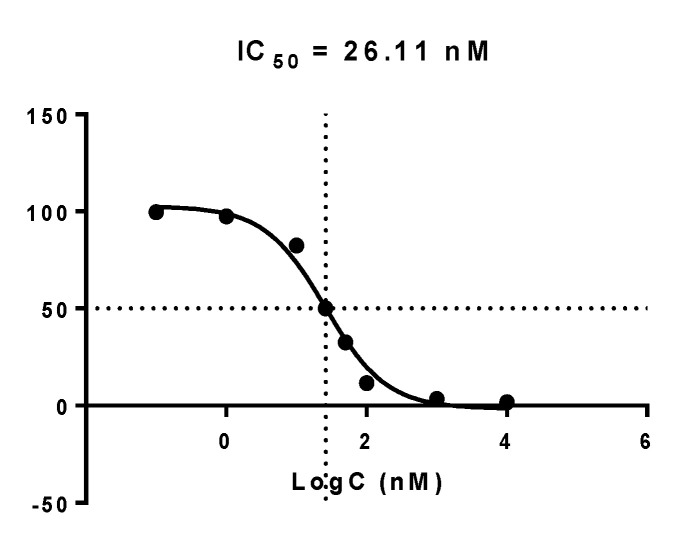
Dose-response curve of **5b** was used for determining the IC_50_ value (nM) after Western blotting analysis.

Complex **5a** was evaluated for its potency to inhibit EGFR phosphorylation in various concentrations by immunoblotting in intact A431 cells that overexpress EGFR (Figure 3). In addition, the percentage of EGFR phosphorylation was calculated, and a dose-response curve was performed (Figure 4). The IC_50_ value of the complex indicating the concentration (nM) needed to inhibit phosphorylation by 50% was 26.11 nM.

The chelator on complex **5a** does not affect the biologically active quinazoline pharmacophore and can cross the cell membrane of A431 cells, extensively inhibiting the EGFR phosphorylation and cell growth. It is also worth mentioning that the metal tricarbonyl core does not significantly modify biological activity. Remarkably, complex **5a** presents the highest inhibition of EGFR phosphorylation compared to previously reported [10,11,13] similar complexes (Table 3), revealing an overall improvement in their design and indicating that elongation of the spacer between the pharmacophore and the chelator was beneficial for the inhibition activity.

### 2.5. Biodistribution Studies

The pharmacokinetic profile of complexes **5b** and **7b** was evaluated in Swiss Albino healthy male mice at 5, 60, and 240 min post intravenous injection. Table 4 presents the results of the biodistribution studies expressed as % Injected Dose (ID)/g ± Standard Deviation (±SD). The two complexes demonstrated fast blood, muscle, lung, and heart clearance. The high lipophilicity characterizing both complexes justifies the rapid hepatobiliary excretion, given the decreasing activity in the liver and the increasing amount of radioactivity in the intestines. Complex **5b** presented faster clearance and elimination in the intestines at 60 min p.i. 34.05% of the radioactivity excreted, compared to 29.89% at 240 min p.i. of complex **7b**. Radioactivity in the urine remained low in general, however, the lower logD_7.4_ value of complex **7b** explains the relatively higher activity in the urines (7.88 ± 0.71%) as it is slightly more hydrophilic than **5b**. Moreover, the low uptake in the brain indicates almost no blood-brain barrier penetration, although the complexes have favorable physicochemical characteristics. The radioactivity levels in the spleen and stomach were very low, proving that the complexes **5b** and **7b** are stable in vivo and they are not being oxidized to [^99m^Tc][TcO_4_]^-^ and [^99m^Tc]TcO_2_.

## 3. Materials and Methods

All solvents and laboratory chemicals were reagent grade and were used without further purification. The HPLC-grade solvents for high-performance liquid chromatography (HPLC) were degassed by a helium flux before and during use. IR spectra were recorded using the Brucker™ Alpha II™ FT-IR/ATR Spectrometer between 4000–400 cm^−1^. NMR spectra were recorded on a Bruker 250 MHz or 500 MHz Avance DRX spectrometer using the residual solvent peak as a reference. HPLC analysis was performed on a Waters 600 chromatography system coupled to a Waters 2487 Dual l Absorbance detector and a Gabi gamma detector from Raytest. Separations were achieved on a Nucleosil C18 (10 mm, 250 mm × 4 mm) column eluted with a binary gradient system at a 1 mL/min flow rate. Mobile phase A was methanol containing 0.1% trifluoroacetic acid, while mobile phase B was water containing 0.1% trifluoroacetic acid. The elution gradient was 0–1 min 95% B (5% A), followed by a linear gradient to 85% A (15% B) in 8 min. This composition was held for 17 min. After a column wash with 95% A for 10 min, the column was re-equilibrated by applying the initial conditions (95% B) for 10 min prior to the next injection.

[^99m^Tc]NaTcO_4_ was obtained in physiological saline as a commercial ^99^Mo/^99m^Tc generator eluate (Ultra-Technekow™ V4 Generator, Curium Pharma, Petten, Netherlands). 6-Amino-4-[(3-bromophenyl)amino] quinazoline (**1**) [21,22], N-(2-pyridylmethyl)aminoethyl acetate (PAMA) (**3**) [23] and the rhenium precursors ReBr(CO)_5_ and *fac*-[Net_4_]_2_[ReBr_3_(CO)_3_] [24] as well as the radioactive precursor *fac*-[^99m^Tc][Tc(OH_2_)_3_(CO)_3_]^+^ [25] have been synthesized according to the literature methods.

### 3.1. Synthesis

#### 3.1.1. Synthesis of the Ligands

Synthesis of Ν-{4-[(3-bromophenyl) amine]-quinazoline-6-yl}6-bromohexanamide (2): 6-Amino-4-[(3-bromophenyl) amino] quinazoline (0.50 g, 1.60 mmol) was dissolved in 10 mL of anhydrous tetrahydrofuran, and the solution was cooled to 0 °C. Next, 6-bromohexanoyl-chloride (0.58 mL, 3.70 mmol) was added dropwise, and the mixture was stirred for 30 min at 0 °C and 3 h at room temperature. Finally, the formed green solid was filtered and washed with tetrahydrofuran. This gave 0.70 g, yield 89%, R_f_ 0.5 in DCM:MeOH (90:10), ^1^H-NMR (500 MHz, DMSO-*d*_6_) δ_H_ 10.26 (s, 1H), 9.92 (s, 1H), 8.71 (s, 1H), 8.53 (s, 1H), 8.14 (s, 1H), 7.91–7.80 (m, 2H), 7.74 (d, *J* = 8.6 Hz, 1H), 7.32 (t, *J* = 7.9 Hz, 1H), 7.26 (d, *J* = 7.4 Hz, 1H), 3.56 (t, *J* = 6.7 Hz, 2H), 2.40 (t, *J* = 7.3 Hz, 2H), 1.89–1.82 (m, 2H), 1.71–1.65 (m, 2H), 1.52–1.43 (m, 2H). ΙR (cm^−1^): 3274 (ΝH_2_), 2934 (CH_2_), 2854 (CH_2_), 1675 (C=O).

Synthesis of 6-(pyridine-2-methyl N, N, acetic ethyl ester-hexanoyl)-4-[(3-bromophenyl) amino]-quinazoline (4): Ν-{4-[(3-bromophenyl) amine]-quinazoline-6-yl}6-bromohexanamide (**2**) (0.2 g, 40.0 mmol) was dissolved in tetrahydrofuran (3 mL) and acetonitrile (8 mL). Sodium iodide (0.18 g, 1.21 mmol) and N, N-diisopropylethylamine (0.27 mL, 1.61 mmol) were added to the solution. PAMA (0.236 g, 1.22 mmol) dissolved in 2 mL of acetonitrile was added dropwise, and the mixture was refluxed for 18 h. After removal of the solvents, product **4** was obtained (0.048 g, yield 20%, R_f_ 0.23 in DCM:MeOH/90:10) by column chromatography purification on silica gel, using a mixture of DCM:MeOH: 25% ammonia (95:5:2) as eluent. ^1^H NMR (500 MHz, DMSO-*d*_6_) δ_H_ 10.21 (s, 1H), 9.88 (s, 1H), 8.71 (s, 1H), 8.56 (s, 1H), 8.45 (d, *J* = 4.1 Hz, 1H), 8.15 (d, *J* = 1.6 Hz, 1H), 7.87–7.81 (m, 2H), 7.76 (d, *J* = 9.0 Hz, 1H), 7.72 (m, 1H), 7.46 (d, *J* = 7.7 Hz, 1H), 7.36–7.3 (m, 1H), 7.28 (d, *J* = 7.2 Hz, 1H), 7.21 (m, 1H), 4.06 (q, *J* = 7.2 Hz), 3.83 (s, 2H), 3.37 (s, 2H), 2.59 (t, *J* = 6.7 Hz, 1H), 2.36 (t, *J* = 6.9 Hz, 1H), 1.63–1.56 (m, 1H), 1.50–1.42 (m, 1H), 1.34–1.28 (m, 1H), 1.17 (t, *J* = 7.0 Hz, 1H). IR (cm^−1^): 2923 (CH_2_), 2853 (CH_2_), 1732 (C=O, ester), 1669 (C=O, amide).

Synthesis of the 6-(cysteine-N-hexanoyl)-4-[(3-bromophenyl)amino]-quinazoline (6): Into a two-neck round-bottomed flask, a mixture of ethanol (10 mL) and sodium bicarbonate 10% (1.2 mL, 2.90 mmol) was vigorously stirred under continuous nitrogen flow for 15 min. *L*-cysteine (0.886 g, 0.73 mmol) and **2** (0.30 g, 0.61 mmol) were added, and the mixture was stirred at room temperature for 18 h under a nitrogen atmosphere. The reaction mixture was diluted with deionized water (10 mL), the pH was adjusted to 10, and the mixture was washed with 30 mL of ethyl acetate. The aqueous layer was concentrated, and the pH was adjusted to 5. The formed precipitate was separated by filtration to obtain **6** as a white solid. This gave 0.239 g, yield 71%, ^1^H-NMR (500 MHz, DMSO-*d*_6_) δ_H_ 10.87 (s, 1H), 10.16 (s, 1H), 8.92 (s, 1H), 8.57 (s, 1H), 8.35 (s, 1H), 8.20 (d, *J* = 8.9 Hz, 1H), 7.96 (d, *J* = 7.9 Hz, 1H), 7.75 (d, *J* = 9.0 Hz, 1H), 7.33 (t, *J* = 8.0 Hz, 1H), 7.27 (d, *J* = 8.0 Hz, 1H), 4.05–3.87 (m, 1H), 3.01 (dd, *J* = 14.4, 3.7 Hz, 1H), 2.94 (dd, *J* = 14.5, 6.8 Hz, 1H), 2.67–2.56 (m, 2H), 2.46–2.35 (m, 2H), 1.75–1.56 (m, 4H), 1.48–1.34 (m, 2H). IR (cm^−1^): 3283 (NH_2_), 2940 (CH_2_), 2845 (CH_2_), 1663 (C=O).

#### 3.1.2. Synthesis of Rhenium Complexes

**Synthesis of rhenium complex [Re(NNO)(CO)_3_] (5a):** Method A. **4** (26.1 mg, 0.04 mmol) dissolved in 4 mL acetonitrile and sodium hydroxide 10% *w/v* (16 µL, 0.04 mmol) were added into a solution of *fac*-[Net_4_]_2_[ReBr_3_(CO)_3_] (30.8 mg, 0.04 mmol) in 4 mL acetonitrile. The reaction mixture was stirred under reflux in an oil bath for 3 h. Then, the reaction mixture was concentrated under a high vacuum, and the green product was purified by column chromatography on silica gel using DCM:MeOH (95:5) as eluent. This gave 24 mg, yield 71%, RP-HPLC t_R_: 14.2 min.

Method B. **4** (30.3 mg, 0.05 mmol) and sodium hydroxide 10% *w/v* (0.02 mL, 0.05 mmol) were added into a solution of [ReBr(CO)_5_] (30.8 mg, 0.04 mmol) in 4 mL acetonitrile. The reaction mixture was stirred under reflux in an oil bath for 18 h. After standing at room temperature, the precipitated green solid was obtained by filtration and recrystallized with acetonitrile. This gave 33 mg, yield 78%, RP-HPLC t_R_: 14.2 min.

^1^H NMR (500 MHz, DMSO-*d*_6_) δ_H_ 10.30 (s, 1H), 9.90 (s, 1H), 8.76 (s, 1H), 8.75 (s, 1H), 8.57 (s, 1H), 8.19–8.11 (m, 2H), 7.89–7.84 (m, 2H), 7.78 (d, *J* = 8.9 Hz, 1H), 7.70 (d, *J* = 8.3 Hz, 1H), 7.58 (t, *J* = 6.5 Hz, 1H), 7.34 (t, *J* = 8.0 Hz, 1H), 7.28 (d, *J* = 7.8 Hz, 1H), 4.76 (d, *J* = 15.8 Hz, 1H), 4.55 (d, *J* = 15.7 Hz, 1H), 3.83 (d, *J* = 16.8 Hz, 1H), 3.57–3.44 (m, 2H), 3.41 (d, *J* = 16.8 Hz, 1H), 2.46 (t, *J* = 7.2 Hz, 2H), 1.86–1.77 (m, 2H), 1.77–1.68 (m, 2H), 1.45–1.36 (m, 2H). ΙR (cm^−1^): 2933 (CH_2_), 2865 (CH_2_), 2024 και 1887 (*fac-*Re(CO)_3_), 1646 (C=O), 1599, 1567, 769, 668.

Synthesis of rhenium complex [Re(SNO)(CO)_3_] (**7a**): Method A. **6** (25.0 mg, 0.05 mmol)), dissolved in 4 mL acetonitrile, and sodium hydroxide 10% *w/v* (18.8 µL, 0.05 mmol) were added into a solution of *fac*-[Net_4_]_2_[ReBr_3_(CO)_3_] (36.2 mg, 0.05 mmol) in 4 mL acetonitrile. The reaction mixture was stirred under reflux in an oil bath for 3 h. Then, the reaction mixture was concentrated under a high vacuum, and the white product was purified by column chromatography on silica gel using DCM:MeOH (95:5) as eluent. This gave 27 mg, yield 69%, RP-HPLC t_R_: 13.9 min.

Method B. **6** (53.3 mg, 0.1 mmol) and sodium hydroxide 10% *w/v* (40 µL, 0.1 mmol) were added into a solution of [ReBr(CO)_5_] (40.0 mg, 0.1 mmol) in 4 mL acetonitrile. The reaction mixture was stirred under reflux in an oil bath for 18 h. Then, the reaction mixture was concentrated under a high vacuum, and the white product was purified by column chromatography on silica gel using DCM:MeOH (95:5) as eluent. This gave 41 mg, yield 52%, RP-HPLC t_R_ = 13.9 min.

^1^H NMR (500 MHz, DMSO-*d*_6_) δ_H_ 10.29 (s, 1H), 9.90 (s, 1H), 8.73 (s, 1H), 8.56 (s, 1H), 8.17 (s, 1H), 7.86 (d, *J* = 8.0 Hz, 2H), 7.77 (d, *J* = 8.8 Hz, 1H), 7.34 (t, *J* = 8.0 Hz, 1H), 7.28 (d, *J* = 7.7 Hz, 1H), 6.19–6.11 (m, 1H), 4.95–4.84 (m, 1H), 4.27–3.99 (m, 2H), 3.94–3.60 (m, 1H), 2.92–2.68 (m, 2H), 2.42 (t, *J* = 6.7 Hz, 2H), 1.73–1.66 (m, 4H), 1.53–1.43 (m, 2H). ΙR (cm^−1^): 2927 (CH_2_), 2824 (CH_2_), 2023 και 1881 (*fac-*Re(CO)_3_), 1662 (C=O).

### 3.2. In Vitro Biological Studies

Cell line. The in vitro biological studies were established using the human epidermoid vulvar carcinoma A431 cell line, which overexpresses EGFR. Cells were available in our laboratory from previous studies [10,11,13]. Cells were maintained in high glucose D-MEM medium with L-glutamine (PAA Laboratories GmbH, Pasching, Austria) and supplemented with 10% fetal bovine serum (PAA Laboratories GmbH) and penicillin/streptomycin (PAA Laboratories GmbH) 100 UI/100 μg per mL, in 5% CO_2_ incubator at 37 °C.

#### 3.2.1. In Vitro Growth Inhibition Assay

The in vitro cytotoxicity of the synthesized ligands and their rhenium complexes was determined by the MTT method. The A431 cells were seeded in 96-well plates at a density of 4000 cells per 100 μL per well and incubated for 24 h so that they were attached to the wells. After 24 h, the studied derivatives were dissolved in DMSO (100 nM) and further diluted in D-MEM in various concentrations. The derivatives were added to the wells in final concentrations of 1 mM, 100 µM, 10 µM, 1 µM, 100 nM, and 10 nM, where the final concentration of DMSO did not exceed 1%. The six concentrations of each derivative and control existed quadrupled into the experiment. The derivatives and the cells were incubated for 72 h. After three days, the medium was removed, and the cells were incubated for 4 h in the presence of MTT (1 mg/mL 3-[4,5-dimethylthiazol-2-yl]-2,5-diphenyltetrazolium bromide, Sigma, Saint Louis, MO, USA) in RPMI (PAA Laboratories GmbH, Pasching, Austria) without phenol red for 2 h at 37 °C. The MTT solution was removed, and 2-propanol was added to each well to stop the cleavage of the tetrazolium ring by dehydrogenase enzymes which convert MTT to an insoluble purple formazan in living cells. The plates were then agitated at room temperature for about 15–20 min, and the level of the colored formazan derivative was determined by measuring optical density (OD) on an ELISA reader (540/620 nm). The results, expressed as the mean of the OD of various replicates *100/OD of the control, were plotted against the corresponding compound concentration in a semi-log chart, and the IC_50_ was determined from the dose-response curve. Cell proliferation assay was performed three times for each compound.

#### 3.2.2. Inhibition of Phosphorylation

The inhibition of EGFR phosphorylation was studied as described in the literature [13]. A431 cells (5 × 10^5^ cells/well) were seeded in 6-well plates and incubated for 24 h. Then, the medium was replaced with fresh medium without fetal bovine serum. The next day, cells were incubated in the presence of the compound at the following concentrations: 0.1 nM, 1 nM, 10 nM, 50 nM, 100 nM, 1 μΜ, and 10 μΜ for 2 h, and then EGF was added (20 ng/mL) and left for 5 min. Two control samples were included in the experiment containing cell cultures without the compound and with or without EGF as a positive and negative control, respectively. Cells were lysed with cell lysis buffer (50 mM Tris-HCl pH 7.4, 150 mM, NaCl, 1 % Triton X-100, 0.25 % *w/v* sodium deoxycholate, 0.1 % *w/v*, sodium dodecyl sulfate, protease, and phosphatase inhibitors with EDTA). Ten micrograms of total protein for each sample were loaded onto polyacrylamide gel (8%) for electrophoresis. Subsequently, the proteins were separated by electrophoresis and transferred to a nitrocellulose membrane. Next, the membrane was washed three times in TBS-T buffer (tris-buffered saline and Tween 20), blocked for one hour in TBS-T with 5% milk (1% fat), and incubated for two hours minimum in a phosphotyrosine antibody (p-Tyr 100, mouse mAb) diluted 1/1000. Afterward, the membrane was washed three times in TBS-T and incubated in an anti-mouse IgG (HRP Linked) antibody diluted 1/1000 for 1 h. Then, the membrane was rewashed three times in TBS-T. The detection was performed using a chemiluminescent detection system (ECL kit, SignalFire ECL Reagent, Cell Signaling Technology, Auburn, MA, USA) according to the manufacturer’s instructions. Protein band quantification was performed using Image-J software, and the percentage of EGFR phosphorylation inhibition was calculated using GraphPad Prism 9.0 software.

### 3.3. Radiochemistry

#### 3.3.1. Preparation of *fac*-[^99m^Tc][Tc(OH_2_)_3_(CO)_3_]^+^ Precursor

Into a sealed vial containing sodium tetraborate (7.5 mg), sodium carbonate (4.4 mg), and sodium tartrate dihydrate (15.0 mg) purged with CO for 2 min, [^99m^Tc]NaTcO_4_ (370–740 MBq, 1 mL) was added. The mixture was incubated at 95 °C for 20 min. After cooling to room temperature, the pH was adjusted to 5 by adding 0.1N HCl (100–120 μL). The *fac*-[^99m^Tc][Tc(OH_2_)_3_(CO)_3_]^+^ complex formation was checked with RP-HPLC. RP-HPLC t_R_ (γ detector): *fac*-[^99m^Tc][Tc(OH_2_)_3_(CO)_3_]^+^: 5.5 min, [^99m^Tc][TcO_4_]^–^: 3.0 min. Yield > 95%.

#### 3.3.2. Synthesis of ^99m^Tc Complexes, **5b** and **7b**

A freshly prepared solution of the *fac*-[^99m^Tc][Tc(OH_2_)_3_(CO)_3_]^+^ (500 μL, 180–370 ΜΒq) was added to a vial containing a solution of ligand **4** (2 × 10^−2^ M, 5 μL) or **6** (5 × 10^−3^ M, 100 μL) in DMSO. The mixture’s final volume was 1 mL, and the pH was adjusted to 5 when required. The vial was sealed, flushed with N_2_ for 1 min, and heated for 30 min at 70 °C. The reaction was monitored by RP-HPLC. RP-HPLC analysis showed the formation of a main peak for **5b** complex (RP-HPLC t_R_: 14.6 min, yield 85–90%) and a single peak for **7b** complex (RP-HPLC t_R_: 14.2 min, yield 95%).

#### 3.3.3. Stability Studies of ^99m^Tc Complexes, **5b** and **7b**

Stability of ^99m^Tc complexes in the reaction mixture: Samples of the ^99m^Tc complexes’ reaction mixture were stored at room temperature for up to 24 h. At 1, 6, and 24 h intervals, 100 μL of each sample was removed and analyzed by RP-HPLC.

Stability of isolated ^99m^Tc complexes: Purification and isolation of **5b** and **7b** complexes were performed by injection of the reaction mixture into RP-HPLC and isolation of the eluted compounds’ peaks, corresponding to the elution time of each complex. Followingly, 0.1 M PBS buffer solution (900 µL) was added to a vial containing 100 µL (50–120 MBq) of the isolated technetium-99m complex. The sample was incubated at 37 °C for up to 24 h and analyzed by RP-HPLC at 1, 6, and 24 h intervals.

Cysteine and histidine challenge: Histidine (100 µL, 1 × 10^−2^ M) or cysteine (100 µL, 1 × 10^−2^ M) solution in 0.1 M PBS was mixed with 100 µL (50–120 MBq) of the RP-HPLC isolated ^99m^Tc complexes in 0.1 M PBS (800 µL). The mixtures were incubated at 37 °C for up to 24 h and analyzed by RP-HPLC at 1, 6, and 24 h intervals.

#### 3.3.4. Lipophilicity

The distribution coefficient of the **5b** and **7b** complexes was determined with the shake-flask method. The RP-HPLC isolated ^99m^Tc complexes (20 μL, ~75 kBq) were mixed with 2 mL of 1-octanol and 2 mL of phosphate buffer solution (PBS, 0.1 M, pH 7.4) in a centrifuge tube. The mixture was vortexed at room temperature for 2 min and centrifuged at 5000 rpm for 10 min. The radioactivity of each phase (200 μL) was counted in a gamma counter. The distribution coefficient was calculated by dividing the cpm/mL in the 1-octanol phase by those in the buffer, and the results were expressed as logD_7.4_. Each experiment was run in triplicate.

### 3.4. Biodistribution Studies in Mice

All the biodistribution experiments were carried out in compliance with the national laws and European protocols (2010/63/EU) related to the conduct of animal experimentation. Nine healthy Swiss Albino mice (male, 30 ± 3 g), divided into three groups of three, were injected with the HPLC-purified **5b** or **7b** complex in saline with 10% EtOH (0.1 mL, ~75 kBq) through the tail vein. For each group, the animals were sacrificed by cardiectomy under slight ether anesthesia 5, 60, and 240 min after the injection. The organs of interest were excised, weighed, and counted in an automatic gamma counter. The bladder and excreted urine were not weighed. The stomach and intestines were not emptied of food contents prior to radioactivity measurements. The percentage of injected dose per organ (%ID/organ) was calculated by comparison of sample radioactivity to standard solutions containing 10% of the injected dose. The calculation for blood and muscle was based on measured activity, sample weight, and body composition data (considering that blood and muscle comprise 7 and 43% of body weight). The percentage of injected dose per gram (%ID/g) was calculated by dividing the %ID/organ by the weight of the organ or tissue.

## 4. Conclusions

Contributing to the development of new, potent, and distinctive EGFR-TK tumor imaging agents, this work demonstrates the synthesis and biological evaluation of two neutral rhenium (I) tricarbonyl complexes bearing the PAMA (**5a**) or cysteine (**7a**) chelator coupled through a six-carbon chain to the 4-anilinoquinazoline pharmacophore moiety. The in vitro cytotoxicity studies in the A431 cancer cell line of the ligands and the Re complexes have similar results to previously reported compounds. Nonetheless, complex **5a** inhibits EGFR phosphorylation with the lowest IC_50_ value among the already reported complexes.

Labeling of the ligands using the *fac*-[^99m^Tc(OH_2_)_3_(CO)_3_]^+^ precursor resulted in the synthesis of the corresponding ^99m^Tc(I) complexes, **5b** and **7b**, respectively, with high radiochemical yield and purity. Biodistribution experiments presented in vivo stability for both complexes, fast blood clearance, and excretion through the hepatobiliary system. These findings inspire further modifications of the spacer and the chelation systems to reduce the compounds’ lipophilicity and improve the pharmacokinetic profile of developing radiopharmaceuticals for imaging EGFR-positive tumors.

## Figures and Tables

**Table 1:** Retention times (t_R_) of ligands, Re- and ^99m^Tc-complexes, and RCP of ^99m^Tc-complexes in PBS, cysteine, and histidine after 24 h at 37 °C.

Compound	t_R_ (min)	%RCP in PBS ^a^ for 24 h	%RCP in Cysteine ^a^ for 24 h	%RCP in Histidine ^a^ for 24 h
**4**	13.1	-	-	-
**5a**	14.2	-	-	-
**5b**	14.6	99 ± 2	99 ± 1	99 ± 1
**6**	12.8	-	-	-
**7a**	13.9	-	-	-
**7b**	14.2	99 ± 1	95 ± 1	95 ± 2

^a^ The values are presented as mean ± standard deviation (SD) from three independent studies.

**Table 2:** Inhibition of cell growth for quinazoline derivatives from three independent experiments in A431 cancer cell line.

Compound	IC_50_ Values (μM) ± SD ^a^
**1**	2.3 ± 0.3
**4**	25.64 ± 3.87
**5a**	8.85 ± 2.62
**6**	4.12 ± 0.92
**7a**	29.56 ± 3.63

^a^ The values are presented as mean ± standard deviation (SD) from three independent experiments.

**Table 3:** Inhibition of EGFR phosphorylation and inhibition of A431 cell growth of quinazoline derivatives present in the literature [10,11,13].

Compound	Inhibition of Cell Growth IC_50_	Inhibition of Phosphorylation IC_50_
	IC_50_ Values (μM) ± SD	IC_50_ Values (nM) ± SD
**8** 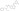	23.8 ± 5.1	-
**9** 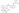	41.2 ± 13.6	756 ± 137
**10** 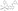	2.0 ± 0.98	114 ± 23
**11** 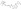	2.9 ± 1.6	108 ± 13
**12** 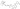	8.24 ± 1.86	70
**13** 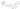	42.51 ± 5.04	-
**14** 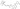	33.29 ± 7.03	-

**Table 4:** Biodistribution data after intravenous injection of *fac*-[^99m^Tc(NNO)(CO)_3_] (**5b**) and *fac*-[^99m^Tc(NNO)(CO)_3_] (**7b**) in healthy mice at 5, 60, and 240 min.

Organ	%Injected Dose/g					
	Complex 5b			Complex 7b		
	5 min	60 min	240 min	5 min	60 min	240 min
Blood	0.85 ± 0.23	0.14 ± 0.03	0.08 ± 0.01	1.06 ± 0.29	0.30 ± 0.09	0.18 ± 0.04
Liver	27.68 ± 2.32	1.24 ± 0.24	0.39 ± 0.08	23.33 ± 3.63	4.88 ± 0.47	4.46 ± 0.16
Heart	0.80 ± 0.12	0.13 ± 0.03	0.05 ± 0.01	0.97 ± 0.29	0.18 ± 0.03	0.12 ± 0.00
Kidneys	8.49 ± 1.33	1.28 ± 0.17	0.06 ± 0.01	13.04 ± 2.34	1.94 ± 0.28	1.12 ± 0.03
Stomach	0.72 ± 0.25	0.64 ± 0.27	0.60 ± 0.18	1.04 ± 0.10	0.97 ± 0.22	0.96 ± 0.06
Intestines	9.22 ± 2.19	34.05 ± 2.05	37.28 ± 3.50	10.62 ± 1.17	26.81 ± 1.61	29.89 ± 2.41
Spleen	0.63 ± 0.09	0.11 ± 0.01	0.07 ± 0.02	0.92 ± 0.32	0.11 ± 0.02	0.09 ± 0.02
Muscle	0.19 ± 0.05	0.08 ± 0.01	0.07 ± 0.07	0.29 ± 0.11	0.13 ± 0.12	0.03 ± 0.00
Lungs	0.97 ± 0.20	0.13 ± 0.03	0.08 ± 0.01	1.19 ± 0.22	0.26 ± 0.04	0.25 ± 0.08
Pancreas	0.63 ± 0.09	0.10 ± 0.03	0.09 ± 0.00	1.10 ± 0.11	0.24 ± 0.05	0.12 ± 0.02
Brain	0.05 ± 0.02	0.03 ± 0.00	0.02 ± 0.00	0.06 ± 0.02	0.02 ± 0.01	0.07 ± 0.06
Urine *	0.09 ± 0.06	1.02 ± 0.17	1.33 ± 0.14	0.08 ± 0.06	5.83 ± 0.02	7.88 ± 0.71

* The results are expressed as % ID. All values are presented as mean ± standard deviation (SD).

## Data Availability

The data presented in this study are available on request from the corresponding author.

## Sample Availability

Samples of the compounds are available from the authors.
